# Noninvasive Assessment of Neuromechanical Coupling and Mechanical Efficiency of Parasternal Intercostal Muscle during Inspiratory Threshold Loading

**DOI:** 10.3390/s21051781

**Published:** 2021-03-04

**Authors:** Manuel Lozano-García, Luis Estrada-Petrocelli, Abel Torres, Gerrard F. Rafferty, John Moxham, Caroline J. Jolley, Raimon Jané

**Affiliations:** 1 Institute for Bioengineering of Catalonia (IBEC), The Barcelona Institute of Science and Technology (BIST), UPC Campus Diagonal-Besòs, Av. d’Eduard Maristany 10–14, 08019 Barcelona, Spain; lestrada@ibecbarcelona.eu (L.E.-P.); atorres@ibecbarcelona.eu (A.T.); rjane@ibecbarcelona.eu (R.J.); 2 Biomedical Research Networking Centre in Bioengineering, Biomaterials and Nanomedicine (CIBER-BBN), 08028 Barcelona, Spain; 3 Department of Automatic Control (ESAII), Universitat Politècnica de Catalunya (UPC)-Barcelona Tech, 08028 Barcelona, Spain; 4 Facultad de Ingeniería, Universidad Latina de Panamá, Panama City 0823-00923, Panama; 5 Centre for Human & Applied Physiological Sciences, School of Basic & Medical Biosciences, Faculty of Life Sciences & Medicine, King’s College London, King’s Health Partners, London SE1 9RT, UK; gerrard.rafferty@kcl.ac.uk (G.F.R.); john.moxham@kcl.ac.uk (J.M.); caroline.jolley@kcl.ac.uk (C.J.J.); 6 King’s College Hospital NHS Foundation Trust, King’s Health Partners, London SE5 9RS, UK

**Keywords:** inspiratory threshold loading, neuromechanical coupling, parasternal intercostal muscles, respiratory pressure, surface electromyography, surface mechanomyography

## Abstract

This study aims to investigate noninvasive indices of neuromechanical coupling (NMC) and mechanical efficiency (MEff) of parasternal intercostal muscles. Gold standard assessment of diaphragm NMC requires using invasive techniques, limiting the utility of this procedure. Noninvasive NMC indices of parasternal intercostal muscles can be calculated using surface mechanomyography (sMMG_para_) and electromyography (sEMG_para_). However, the use of sMMG_para_ as an inspiratory muscle mechanical output measure, and the relationships between sMMG_para_, sEMG_para_, and simultaneous invasive and noninvasive pressure measurements have not previously been evaluated. sEMG_para_, sMMG_para_, and both invasive and noninvasive measurements of pressures were recorded in twelve healthy subjects during an inspiratory loading protocol. The ratios of sMMG_para_ to sEMG_para_, which provided muscle-specific noninvasive NMC indices of parasternal intercostal muscles, showed nonsignificant changes with increasing load, since the relationships between sMMG_para_ and sEMG_para_ were linear (R^2^ = 0.85 (0.75–0.9)). The ratios of mouth pressure (P_mo_) to sEMG_para_ and sMMG_para_ were also proposed as noninvasive indices of parasternal intercostal muscle NMC and MEff, respectively. These indices, similar to the analogous indices calculated using invasive transdiaphragmatic and esophageal pressures, showed nonsignificant changes during threshold loading, since the relationships between P_mo_ and both sEMG_para_ (R^2^ = 0.84 (0.77–0.93)) and sMMG_para_ (R^2^ = 0.89 (0.85–0.91)) were linear. The proposed noninvasive NMC and MEff indices of parasternal intercostal muscles may be of potential clinical value, particularly for the regular assessment of patients with disordered respiratory mechanics using noninvasive wearable and wireless devices.

## 1. Introduction

Evaluating respiratory muscle function contributes to enhancing the diagnosis, phenotyping, and monitoring of patients with respiratory symptoms and neuromuscular diseases [1]. Inspiratory pressure generation is dependent on the level of muscle electrical activation and the transduction of this electrical activation into pressure, termed neuromechanical coupling (NMC). Poor transduction of inspiratory muscle electrical activation into pressure, neuromechanical uncoupling, is common in obstructive lung disease [2,3,4], and has been related to the perception of dyspnea in both obstructive lung disease [5] and in neuromuscular disease [6]. Measuring inspiratory muscle NMC is therefore clinically important for the assessment of patients with disordered respiratory mechanics.

Inspiratory muscle NMC estimation requires simultaneous measurement of muscle electrical activation and mechanical output. To date, most studies reporting inspiratory muscle NMC have focused on the diaphragm, the principal inspiratory muscle [7]. The gold standard measurement of diaphragm electrical activation and mechanical output involves the use of invasive techniques, including crural diaphragm electromyography using a multipair esophageal electrode catheter (oesEMG_di_) to assess neural respiratory drive [8], and the balloon-catheter technique to measure transdiaphragmatic pressure (P_di_) [1]. However, the utility of these techniques is limited by the invasive nature of the measurements, subject tolerance, and requirement for specialist equipment and training. In this regard, noninvasive measurement of inspiratory muscle NMC would facilitate the assessment of respiratory muscle function.

Parasternal intercostal muscles are obligatory muscles of inspiration and their activation is coupled to that of the diaphragm [9]. Measurements of surface electromyography of the parasternal intercostal muscles (sEMG_para_) have been shown to provide a robust measure of load on the respiratory muscle pump and to strongly correlate with measurements of oesEMG_di_ [10,11,12]. Measurements of sEMG_para_ have also been related to breathlessness in both healthy subjects [3,12] and patients with respiratory disease [3,12,13,14,15]. Therefore, and given the advantage of being noninvasive, sEMG_para_ has been proposed as an alternative measure for the assessment of neural respiratory drive [10,11].

Surface mechanomyography (sMMG) measures muscle surface vibrations due to motor unit mechanical activity [16], and represents the mechanical counterpart of motor unit electrical activity, as measured by surface electromyography (sEMG). In non-respiratory skeletal muscles, the ratio of sMMG to sEMG has been proposed for the assessment of muscle electromechanical efficiency [17,18]. In the respiratory system, sMMG and pressure represent different mechanical states of inspiratory muscles, the latter being a more global measure of respiratory muscle mechanical output. We have recently used sMMG and sEMG measurements from lower chest wall intercostal spaces (sMMG_lic_ and sEMG_lic_, respectively) to provide noninvasive indices of lower chest wall inspiratory muscle NMC [19], which did not change significantly with increasing inspiratory load due to the linear relationship between sMMG_lic_ and sEMG_lic_ measurements. Measurements of sMMG_lic_ have also been used, in combination with measurements of mouth pressure (P_mo_), to calculate the mechanical efficiency (MEff) of lower chest wall inspiratory muscles, that is the transformation of muscle mechanical activation into pressure generation, in healthy subjects and patients with chronic obstructive pulmonary disease (COPD) [20]. Lower chest wall recordings are, however, less easily accessible and more contaminated by non-respiratory chest wall and abdominal muscle activation than parasternal intercostal recordings [21,22]. Nevertheless, the use of parasternal intercostal muscle sMMG (sMMG_para_) as a noninvasive measure of inspiratory muscle mechanical output has not previously been investigated.

Inspiratory muscle mechanical output is usually estimated as pressure. Gold standard P_di_ measurements are derived from simultaneous measurements of gastric (P_gas_) and esophageal (P_oes_) pressures (P_di_ = P_gas_ – P_oes_). Since P_di_ is specific to the diaphragm, P_di_ has been widely used, together with oesEMG_di_, for the assessment of diaphragm NMC [4,12,19,23,24,25,26]. Measurements of P_oes_ alone are, however, not specific to the diaphragm and are influenced by the activation of all inspiratory muscles [1]. Compared to P_di_, P_oes_ is therefore expected to better reflect the mechanical output of extra-diaphragmatic muscles, including the parasternal intercostal muscles. Moreover, P_mo_, which provides a noninvasive approximation of P_oes_ [1], could be used to obtain noninvasive indices of NMC and MEff of parasternal intercostal muscles. However, to our knowledge, no study has evaluated the relationships among simultaneous invasive and noninvasive measurements of respiratory pressures and both sEMG_para_ and sMMG_para_ measurements for the assessment of NMC and MEff of parasternal intercostal muscles.

The principal aim of this study was therefore to investigate the use of sMMG_para_ as a novel noninvasive index of parasternal intercostal muscle mechanical output. In addition, we wished to examine the relationship between sMMG_para_ and sEMG_para_ to provide muscle-specific noninvasive indices of NMC of parasternal intercostal muscles, in healthy subjects during an incremental inspiratory threshold loading protocol. We hypothesized that, as in lower chest wall inspiratory muscles, there would be a linear relationship between measurements of sMMG_para_ and sEMG_para_ in healthy subjects, thus resulting in nonsignificant changes in NMC of parasternal intercostal muscles (the ratio of sMMG_para_ to sEMG_para_ measurements) during threshold loading. We also aimed to explore the relationship between measurements of sEMG_para_ and sMMG_para_ and both invasive and noninvasive measurements of respiratory pressures, to obtain indices of NMC and MEff of parasternal intercostal muscles.

## 2. Materials and Methods

### 2.1. Data Acquisition and Preprocessing

#### 2.1.1. Measurements

This study was carried out in the Respiratory Physiology Laboratory at the King’s College London, King’s College Hospital, London, United Kingdom. Ethics approval was obtained from the National Health Service Health Research Authority (National Research Ethics Service Committee London—Dulwich 05/Q0703) and the study was conducted in accordance with the Declaration of Helsinki.

All recordings were obtained from 12 healthy subjects, including 6 male and 6 female, with no medical history of neuromuscular or cardiorespiratory disease and with the following characteristics (median (interquartile range)): age 33 (30–39) years, body mass index (BMI) 22.2 (20.6–24.2) kg/m^2^, forced expiratory volume in 1 second 98% (95–106%) % of predicted, and forced expiratory volume in 1 second/forced vital capacity 82% (74–84%) % of predicted [19]. Written consent was provided by all subjects prior to study participation.

sEMG_para_ was acquired using a pair of surface electrodes in bipolar configuration located bilaterally in the second intercostal space [10,27]. The sEMG_para_ recordings were amplified, high-pass filtered with a cut-off frequency of 10 Hz, and alternating current coupled. sMMG_para_ was acquired using a triaxial accelerometer (TSD109C2; BIOPAC Systems Inc, Goleta, CA, USA) attached to the skin in the second intercostal space, between the sEMG_para_ electrodes and close to the right one. P_gas_ and P_oes_ were acquired using a catheter tip pressure transducer (CTO-2; Gaeltec Devices Ltd., Dunvegan, UK), as previously described [12]. Airflow was acquired using a pneumotachometer (4830; Hans Rudolph Inc, Shawnee, KS, USA). P_mo_ was acquired using a differential pressure transducer (MP45; Validyne Engineering, Northridge, CA, USA) connected to a side port of the pneumotachometer.

All respiratory signals were acquired using a 16-bit analog-to-digital converter (PowerLab 16/35; ADInstruments Ltd, Oxford, UK) at a sampling frequency of 4000 Hz (sEMG_para_), 2000 Hz (sMMG_para_), and 100 Hz (pressures and airflow). LabChart software (Version 7.2, ADInstruments Pty, Colorado Springs, CO, USA) was used to manage the signal acquisition.

#### 2.1.2. Acquisition Protocol

Initially, each participant carried out two types of maximal volitional respiratory maneuvers: maximal static inspiratory pressure (PImax) [1] and maximal inspirations to total lung capacity [10,28]. These maneuvers were performed several times to ensure each subject’s maximal volitional effort. All signals were recorded during two minutes of resting breathing (L0). After that, each participant carried out an inspiratory threshold loading protocol at inspiratory threshold loads ranging from 12% (L1) to 60% (L5) of PImax, in increments of 12%, as we previously described [19]. Inspiratory loads were generated using an electronic inspiratory muscle trainer (POWERbreathe K5; POWERbreathe International Ltd, Southam, UK) attached to the distal end of the pneumotachometer. Subjects breathed through the pneumotachometer and performed 30 breaths against each inspiratory load, with a resting period in between to allow all measurements to return to baseline.

#### 2.1.3. Data Preprocessing

All data were processed and analyzed in MATLAB (The MathWorks, Inc., vR2020a, Natick, MA, USA). sEMG_para_ signals were down-sampled at 2000 Hz and filtered between 10 and 600 Hz. Removal of 50 Hz interference was performed by means of a comb filter. sMMG_para_ signals were down-sampled at 500 Hz and filtered between 5 and 40 Hz. The three acceleration components recorded by the accelerometer were root sum squared to calculate the magnitude of the sMMG_para_ signal (|sMMG_para_|).

A zero-crossing threshold-based detector was applied to P_mo_ in order to detect inspiratory and expiratory segments during resting breathing and each threshold load for subsequent inspiratory muscle activity estimation. For each respiratory cycle, the following parameters were calculated: inspiratory time, inspiratory time/total respiratory cycle time, inspiratory volume (i.e., the area under the curve of the inspiratory flow), and breathing frequency. The median values of all respiratory cycles were then calculated separately for L0–L5, and 10 respiratory cycles that contained the four parameters nearest to the median values were automatically selected, resulting in 60 respiratory cycles per subject.

The moving minimum of the P_di_ signal and the moving maximum of the P_oes_ signal were calculated using a moving window of 1.5 times the maximum inspiratory time of each load, and subtracted from the P_di_ and P_oes_ signals, respectively.

### 2.2. Data Processing

#### 2.2.1. Inspiratory Muscle Activity Estimation

Inspiratory muscle activity estimation has usually been based on conventional amplitude estimators, such as the average rectified value (ARV) or the root mean square (RMS) [28,29]. However, when applied to myographic respiratory signals, these parameters are greatly affected by cardiac activity, and therefore prior rejection of signal segments that contain cardiac noise is required. Fixed sample entropy (fSampEn) has been demonstrated to reduce cardiac activity in myographic respiratory signals [30,31], allowing inspiratory muscle activity to be estimated without the need for prior rejection of cardiac artefacts. fSampEn consists in calculating sample entropy within a moving window over a signal, using a fixed tolerance value for all windows [30]. In this way, fSampEn is sensitive to variations in both signal complexity and signal amplitude. Therefore, like ARV and RMS, fSampEn can track amplitude variations in myographic respiratory signals. Furthermore, since cardiac artifacts exhibit a more deterministic pattern compared to respiratory EMG and MMG signals, fSampEn also inherently reduces cardiac activity, and therefore it is less influenced by cardiac artefacts than ARV or RMS.

In this study, fSampEn was calculated for the sEMG_para_ (fSEsEMG_para_) and |sMMG_para_| (fSE|sMMG_para_|) signals, using the optimal fSampEn parameters that we previously described for respiratory muscle activity estimation (i.e., a moving window of 500 ms with a 50 ms step, *m* equal to 2, and *r* equal to 0.3 (sEMG_para_) and 0.5 (|sMMG_para_|) times the global standard deviation of each signal) [32]. Inspiratory muscle activity was then estimated for each respiratory cycle as the mean inspiratory P_di_, P_oes_, P_mo_, fSE|sMMG_para_|, and fSEsEMG_para_. The mean values of fSE|sMMG_para_| and fSEsEMG_para_ were expressed as percentages of their respective largest mean values obtained during the inspiratory threshold loading protocol and the two maximal volitional maneuvers (fSE|sMMG_para_|_%max_ and fSEsEMG_para%max_).

#### 2.2.2. Neuromechanical Coupling and Mechanical Efficiency

NMCs of parasternal intercostal muscles were calculated as the ratios of fSE|sMMG_para_|_%max_, mean P_di_, mean P_oes_, and mean P_mo_ to fSEsEMG_para%max_ (NMC_MMG-EMGpara_, NMC_Pdi-EMGpara_, NMC_Poes-EMGpara_, and NMC_Pmo-EMGpara_, respectively). MEffs of parasternal intercostal muscles were calculated as the ratios of mean P_di_, mean P_oes_, and mean P_mo_ to fSE|sMMG_para_|_%max_ (MEff_Pdi-MMGpara_, MEff_Poes-MMGpara_, and MEff_Pmo-MMGpara_, respectively). The average value of the ten respiratory cycles selected for resting breathing and each load was calculated for all NMCs and MEffs.

### 2.3. Statistical Analysis

All data correspond to median and interquartile range. Changes in respiratory pressures, fSE|sMMG_para_|_%max_, and fSEsEMG_para%max_ with increasing threshold load were assessed using Friedman tests followed by multiple Wilcoxon signed-rank tests with Bonferroni adjusted *p*-values.

An increasing NMC with increasing load can be well explained by an exponential relationship between the measurements involved [19]. By contrast, a NMC that remains almost constant or increases slightly with increasing load indicates that the relationship between the measurements involved can be well explained by a linear model. Therefore, in this study, the relationships between measurements of respiratory pressures, fSE|sMMG_para_|_%max_, and fSEsEMG_para%max_ were assessed individually using both linear and exponential regression models, together with Spearman’s rank correlation coefficients (*ρ*). Moreover, changes in NMCs and MEffs during threshold loads L1–L5 were assessed using Friedman tests, followed by multiple Wilcoxon signed-rank tests with Bonferroni adjustment for multiple comparisons, with the same method that we previously described [19]. The significance level for all statistical tests was set at 0.05.

## 3. Results

**Figure 1:**
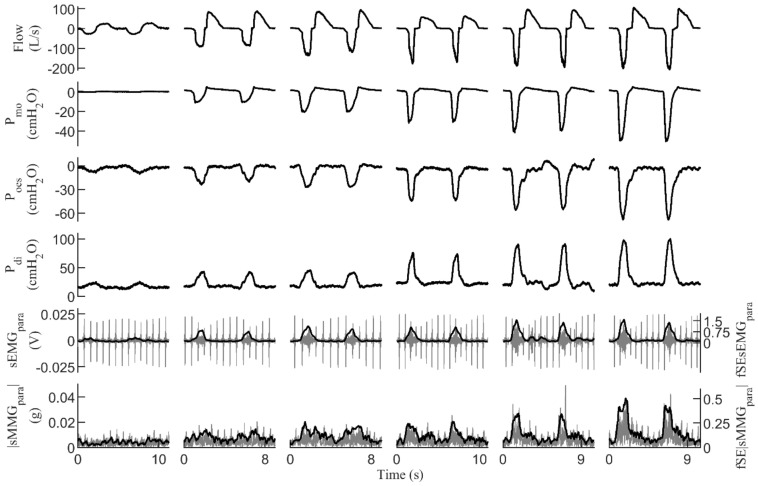
Signals recorded during the inspiratory threshold loading protocol in a healthy subject. Two respiratory cycles are shown for quiet resting breathing and inspiratory threshold loads at 12%, 24%, 36%, 48%, and 60% of maximal static inspiratory pressure. For the sEMG_para_ and |sMMG_para_| signals, the corresponding fixed sample entropy time-series (fSEsEMG_para_ and fSE|sMMG_para_| respectively) are also shown. P_mo_ = mouth pressure, P_oes_ = esophageal pressure, P_di_ = transdiaphragmatic pressure, sEMG_para_ = surface electromyography of the parasternal intercostal muscles, |sMMG_para_| = surface mechanomyography of the parasternal intercostal muscles.

Figure 1 shows an example of respiratory signals recorded in a healthy subject during resting breathing and the inspiratory threshold loading protocol.

### 3.1. Respiratory Pressures

**Figure 2:**
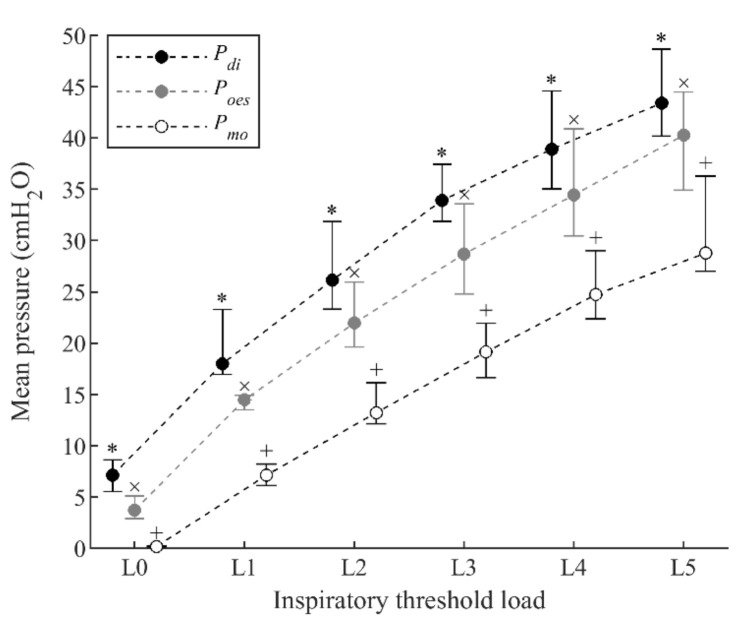
Evolution of respiratory pressures during progressive inspiratory threshold loading. Data points represent median and interquartile (IQR) range of the 12 subjects for each load. All data points with the same symbol (*, ×, or +) were significantly different to each other.

Median (interquartile range (IQR)) PImax for the group was 87.0 (78.0–116.5) cmH_2_O. The inspiratory threshold loads increased from 10.5 (9.5–14.0) cmH_2_O during load L1 to 52.0 (47.0–70.0) cmH_2_O during load L5. Mean P_di_, mean P_oes_, and mean P_mo_ increased significantly between successive loads during the inspiratory threshold loading protocol (Figure 2). Although all pressures increased in parallel during threshold loading, mean P_mo_ was consistently lower than mean P_oes_ and mean P_di_.

### 3.2. sMMG and sEMG of Parasternal Intercostal Muscles during Threshold Loading

**Figure 3:**
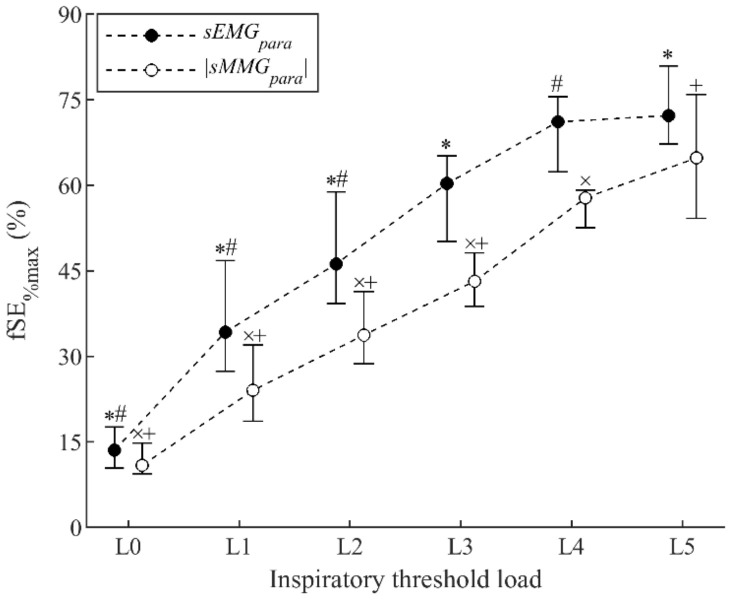
Fixed sample entropy measurements of surface mechanomyography (fSE|sMMG_para_|_%max_) and surface electromyography (fSEsEMG_para%max_) of the parasternal intercostal muscles during inspiratory threshold loading. Data points represent median and interquartile range of the 12 subjects for each load. All data points with the same symbol (*, #, ×, or +) were significantly different to each other.

Measurements of fSE|sMMG_para_|_%max_ increased progressively from 10.9% (9.4–14.8%) during resting breathing to 64.7% (54.2–75.9%) during load L5 (Figure 3). Similarly, measurements of fSEsEMG_para%max_ increased from 13.5% (10.4–17.6%) during resting breathing to 72.2% (67.2–80.9%) during load L5. Increases in fSE|sMMG_para_|_%max_ and fSEsEMG_para%max_ were statistically significant between successive loads, except between loads L3 and L4 in fSEsEMG_para%max_, and between loads L4 and L5 in both fSE|sMMG_para_|_%max_ and fSEsEMG_para%max_ measurements. Moreover, although fSE|sMMG_para_|_%max_ and fSEsEMG_para%max_ increased in parallel during threshold loading, fSE|sMMG_para_|_%max_ was consistently lower than fSEsEMG_para%max_.

### 3.3. Noninvasive Measurements of Neuromechanical Coupling and Mechanical Efficiency of Parasternal Intercostal Muscles

Having an increasing or almost constant pattern of NMC and MEff indices with increasing load depends on whether the relationship between the measurements involved is exponential or linear respectively, as we previously described [19]. Accordingly, the relationships between measurements of respiratory pressures, fSE|sMMG_para_|_%max_, and fSEsEMG_para%max_ were firstly assessed individually using both linear and exponential regression models. Secondly, the evolution of the group NMC and MEff indices during threshold loads L1–L5 was assessed using Friedman tests, followed by multiple Wilcoxon signed-rank tests with Bonferroni adjustment for multiple comparisons.

**Figure 4:**
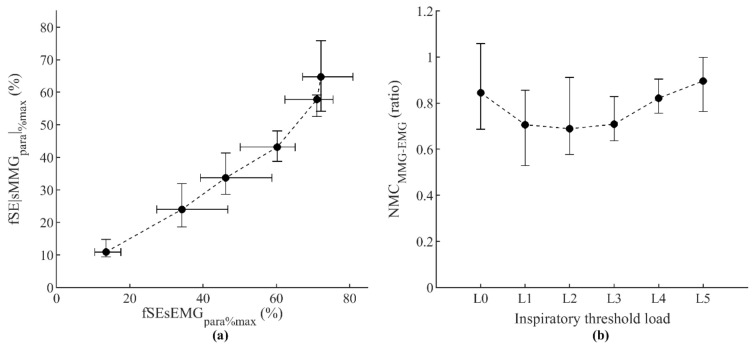
Relationship between fixed sample entropy measurements of surface mechanomyography (fSE|sMMG_para_|_%max_) and surface electromyography (fSEsEMG_para%max_) of the parasternal intercostal muscles (**a**) and the corresponding neuromechanical coupling ratio (NMC_MMG-EMGpara_) (**b**), during the incremental inspiratory threshold loading protocol. Data points represent median and interquartile range of the 12 subjects for each load.

The relationship between fSE|sMMG_para_|_%max_ and fSEsEMG_para%max_ measurements shown in Figure 4a was firstly assessed. Very strong positive correlations between fSE|sMMG_para_|_%max_ and fSEsEMG_para%max_ were found individually (Table 1). Individual linear and exponential regression results shown in Table 2 indicate that the linear model, besides being simpler, performed slightly better than the exponential model to describe the relationships between fSE|sMMG_para_|_%max_ and fSEsEMG_para%max_. The evolution of the group NMC_MMG-EMGpara_ during progressive inspiratory threshold loading is shown in Figure 4b. Slight and nonsignificant increases were found in NMC_MMG-EMGpara_ between loads L1 and L5, confirming that the relationship between fSE|sMMG_para_|_%max_ and fSEsEMG_para%max_ can be well explained by a linear model.

**Figure 5:**
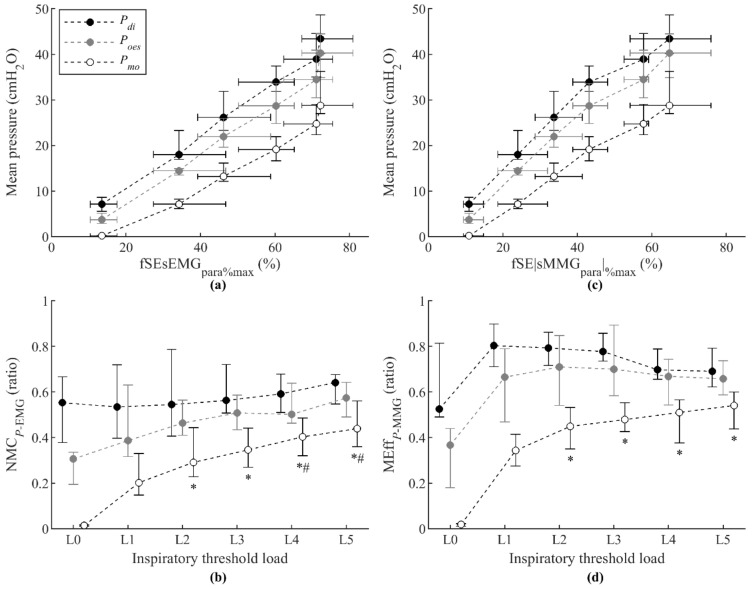
Relationship between respiratory pressures and fixed sample entropy measurements of both surface electromyography (fSEsEMG_para%max_) (**a**) and surface mechanomyography (fSE|sMMG_para_|_%max_) (**c**) of the parasternal intercostal muscles, and the corresponding neuromechanical coupling (NMC_P-EMGpara_) (**b**) and mechanical efficiency (MEff_P-MMGpara_) (**d**) ratios, during the incremental inspiratory threshold loading protocol. Data points represent median and interquartile range of the 12 subjects for each load. Symbols ∗ and # indicate statistically significant differences with respect to inspiratory threshold loads L1 and L2, respectively.

Secondly, the relationships between mean P_di_, mean P_oes_, and mean P_mo_ and fSEsEMG_para%max_ measurements shown in Figure 5a were analyzed. Individual analyses showed very strong positive correlations between mean P_di_, mean P_oes_, and mean P_mo_ and fSEsEMG_para%max_ (Table 1). Moreover, the linear and exponential regression results shown in Table 2 indicated that these relationships are better described by linear models than by exponential models. The group NMC_Pdi-EMGpara_ and NMC_Poes-EMGpara_ increased very little, not significantly, from load L1 to load L5 (Figure 5b). The group NMC_Pmo-EMGpara_ increased significantly from load L1 to load L2, but not significantly from load L3 to load L5, thus tending to stabilize around a constant value as the load increases. These results are consistent with the fact that the relationships between mean P_di_, mean P_oes_, and mean P_mo_ and fSEsEMG_para%max_ are better explained by linear than by exponential models.

Finally, the relationships between mean P_di_, mean P_oes_, and mean P_mo_ and fSE|sMMG_para_|_%max_ measurements shown in Figure 5c were analyzed. Individual analyses showed very strong positive correlations between mean P_di_, mean P_oes_, and mean P_mo_ and fSE|sMMG_para_|_%max_ (Table 1). The linear and exponential regression results indicated that these relationships are better described by linear models than by exponential models (Table 2). Accordingly, the group MEff_Pdi-MMGpara_ and MEff_Poes-MMGpara_ changed very little, not significantly, from load L1 to load L5 (Figure 5d), and the group MEff_Pmo-MMGpara_ increased significantly from load L1 to load L2, but not significantly from load L2 to load L5, thus tending to change little as the load increases.

## 4. Discussion

This study describes for the first time the use of sMMG_para_ as a measure of the mechanical activity of parasternal intercostal muscles, allowing calculation of noninvasive indices of NMC of parasternal intercostal muscles (NMC_MMG-EMGpara_, i.e., the ratio of fSE|sMMG_para_|_%max_ to fSEsEMG_para%max_). NMC_MMG-EMGpara_ showed little and not significant changes with progressive increases in inspiratory load between 12% and 60% of PImax. This is due to the mostly linear increase in fSE|sMMG_para_|_%max_ relative to fSEsEMG_para%max_ during threshold loading.

The aforementioned results are equivalent to those found in our previous study using sMMG_lic_ and sEMG_lic_ recordings from the lower chest wall inspiratory muscles [19]. In that study, we showed that both sEMG_lic_ and sMMG_lic_ measurements reflect, in part, the activation of the diaphragm, but also extra-diaphragmatic lower chest wall and abdominal muscle activation [22,33,34], which progressively increases with increasing threshold load to optimize the functioning of the diaphragm [35]. The contribution of the activation of extra-diaphragmatic muscles to sMMG_lic_ and sEMG_lic_ in a similar way was the reason for the parallel increase of fSE|sMMG_lic_|_%max_ and fSEsEMG_lic%max_ during incremental threshold loading, and therefore for the nonsignificant changes in lower chest wall inspiratory muscle NMC from load L1 to load L5.

In the present study, both fSE|sMMG_para_|_%max_ and fSEsEMG_para%max_ measurements have also been found to increase progressively and mostly in parallel with increasing threshold load. The increasing pattern of parasternal intercostal muscle activation during inspiratory threshold loading was previously reported by Reilly et al. [10] using sEMG_para_ measurements in healthy subjects. They reported that, although root-mean-square-based measurements of oesEMG_di%max_ were consistently greater than those of sEMG_para%max_, there was a strong relationship between them and they increased mostly linearly during threshold loading. Such coupling between parasternal intercostal muscles and the diaphragm had previously been suggested by De Troyer and Sampson [9], who indicated that parasternal intercostals are involuntarily activated, together with the diaphragm, during inspiratory breathing efforts. Accordingly, sEMG_para_ has been proposed as an alternative noninvasive measure of neural respiratory drive [10,12,14,36]. 

In non-respiratory skeletal muscles, the relationship between measurements of sMMG and sEMG has been used to characterize some neuromuscular diseases. Orizio et al. [37] analyzed the ratio of sMMG to sEMG, which they called electromechanical coupling efficiency, of finger flexors in patients with myotonic dystrophy, who presented lower values as compared to those of control subjects. Barry et al. [38] used the ratio of sMMG to sEMG, recorded from the biceps brachii, to study several pediatric muscle diseases. They found a significant reduction in the ratio in affected subjects. The same ratio was used in patients with spastic cerebral palsy by Akataki et al. [39], who found significantly lower ratios of sMMG to sEMG in the patients than in the normal group.

The sMMG signal provides information about muscle contractile properties, reflects the mechanical properties of motor unit activity, and serves as an estimate of muscle force generation [16]. Therefore, relationships between sMMG and sEMG measurements, as expressed by NMC_MMG-EMGpara_ in this study, provide muscle-specific noninvasive indices of NMC, which depend mainly on muscle mechanics. In the respiratory system, however, such indices reflect only the first step in the transformation of neural respiratory drive into ventilation. Next steps include the translation of respiratory muscle force into pressure, and the translation of pressure into ventilation. These steps depend on several aspects, such as chest wall geometry, airways resistance, or lung compliance, which can be altered in patients with disordered ventilatory mechanics, such as in COPD or in restrictive lung disease, thus causing neuromechanical and neuroventilatory uncoupling [40]. Therefore, it is desirable to have noninvasive indices of NMC, other than NMC_MMG-EMGpara_, capable of reflecting the uncoupling that may occur in the different steps of the transformation of neural respiratory drive into ventilation. Different combinations of measurements of the electrical activation of respiratory muscles, respiratory pressures, and lung volumes have been proposed in previous studies as indices of NMC and neuroventilatory coupling [2,6,19,41,42,43]. However, the indices proposed in those previous studies involved at least one invasive measurement (i.e., P_oes_, P_di_, or oesEMG_di_).

This is the first study to explore the relationships between measurements of sEMG_para_ and sMMG_para_, and measurements of P_di_, P_oes_, and P_mo_ to propose noninvasive indices of NMC and MEff of parasternal intercostal muscles. We found progressive and mostly linear increases in fSEsEMG_para%max_ relative to mean P_di_, mean P_oes_, and mean P_mo_ during threshold loading. Analogous results were found between fSEsEMG_lic%max_ and P_di_, expressed as a percentage of maximum, in our previous study [19]. Respiratory pressures are considered measurements of global respiratory mechanical output [1], thus reflecting the action of several inspiratory muscles. The linear relationships found between fSEsEMG_para%max_ and the three different mean pressures (mean P_di_, mean P_oes_, and mean P_mo_) may result therefore from the contribution of progressive activation of parasternal intercostal muscles to sEMG_para_, and of parasternal and other extra-diaphragmatic inspiratory muscles to respiratory pressure measurements. Accordingly, NMC_Pdi-EMGpara_, NMC_Poes-EMGpara_, and NMC_Pmo-EMGpara_ remained almost constant or increased slightly during threshold loading. It is noteworthy, however, that NMC_Pmo-EMGpara_ increased significantly at the onset of inspiratory loading and from load L1 to load L2, which was due to the low values of P_mo_ during quiet resting breathing. Nevertheless, NMC_Pmo-EMGpara_ behaved similarly to NMC_Poes-EMGpara_ and NMC_Pdi-EMGpara_ as load increased. Progressive and mostly linear increases were also found in fSE|sMMG_para_|_%max_ relative to mean P_di_, mean P_oes_, and mean P_mo_ during threshold loading. These results are in accordance with those previously found between mean fSE|sMMG_lic_| and mean P_di_ in our previous study [33]. Accordingly, MEff_Pdi-MMGpara_ and MEff_Poes-MMGpara_ showed nonsignificant changes during threshold loading. MEff_Pmo-MMGpara_, as NMC_Pmo-EMGpara_, increased significantly at the onset of inspiratory loading and from load L1 to load L2, but behaved similarly to MEff_Poes-MMGpara_ and MEff_Pdi-MMGpara_ at higher loads. The NMC_Pmo-EMGpara_ and MEff_Pmo-MMGpara_ indices proposed in this study therefore provide noninvasive measurements of the contribution of parasternal intercostal muscle activation to the generation of respiratory pressures.

Parasternal intercostal recordings have the advantage, over lower chest wall inspiratory muscle recordings, of being less affected by the limitations generally associated with surface recordings, such as the difficulty in finding the optimal sensor position or the strong influence of chest wall thickness and subcutaneous fat [44,45]. Moreover, parasternal intercostal recordings are less susceptible to crosstalk from postural chest wall and abdominal muscle activity [21,22]. The noninvasive indices of NMC_MMG-EMGpara_, NMC_Pmo-EMGpara_, and MEff_Pmo-MMGpara_ proposed in this study would therefore make the evaluation of respiratory muscle function easier and faster to perform, and thus more acceptable in patients with altered respiratory mechanics, such as in obstructive lung disease and neuromuscular disease. Neuroventilatory uncoupling resulting from respiratory muscle weakness and an increased elastic load of the lungs has been related to the degree of dyspnea in patients with neuromuscular disease [6]. Also, in chronic pulmonary diseases, neuromechanical uncoupling has been associated with the perception of breathlessness and limited exercise tolerance [3,5]. The proposed indices would therefore be of potential value to the clinical assessment of these patients.

This study may provide a basis for future research. The clinical utility of the proposed noninvasive NMC and MEff indices requires testing in disease states, since our study dataset was recorded from twelve healthy subjects. The size of the dataset reflects the difficulty in recruiting subjects for studies using invasive measures of diaphragmatic function. The study participants had body mass index values within the normal range. However, it is well known that sEMG and sMMG are affected by the thickness of subcutaneous fat [46,47]. The effect of body mass index on sMMG_para_ and sEMG_para_ measurements should therefore be a focus of future investigation.

## 5. Conclusions

We have proposed the combined use of P_mo_ and parasternal intercostal sEMG and sMMG recordings to obtain noninvasive indices of NMC and MEff of parasternal intercostal muscles, in healthy adults during an incremental inspiratory threshold loading protocol. The combination of sMMG_para_ and sEMG_para_ measurements (NMC_MMG-EMGpara_) provides a muscle-specific noninvasive index of NMC of parasternal intercostal muscles, whose pattern during threshold loading is similar to that previously found in lower chest wall inspiratory muscle NMC. Global noninvasive indices of NMC and MEff of parasternal intercostal muscles have also been proposed by combining P_mo_ measurements and both sEMG_para_ and sMMG_para_ measurements (NMC_Pmo-EMGpara_ and MEff_Pmo-MMGpara_, respectively), reflecting the contribution of parasternal intercostal muscles to global respiratory mechanical output. Similar patterns have been found in NMC_Pmo-EMGpara_ and MEff_Pmo-MMGpara_, and their analogous invasive indices, calculated using P_oes_ and P_di_.

The proposed noninvasive indices derived from P_mo_, sMMG_para_, and sEMG_para_ may prove to be useful indices of NMC and MEff of parasternal intercostal muscles, particularly for the assessment of respiratory muscle function using wearable devices. Advances in sensor technologies have led to an increasing trend and interest in the use of wearable and wireless physiological monitoring devices in medicine [48,49,50]. These devices may contribute to improving the assessment of patients with chronic respiratory diseases by allowing home monitoring of respiratory muscle function in a wireless and noninvasive manner. In this regard, the proposed noninvasive indices of NMC could be easily implemented in a portable device capable of acquiring sEMG and sMMG signals, allowing regular monitoring of patients with impaired respiratory mechanics.

## Figures and Tables

**Table 1:** Spearman’s ρ between measurements of respiratory pressures, fSE|sMMG_para_|_%max_, and fSEsEMG_para%max_.

ID	fSEsEMG_para%max_ fSE|sMMG_para_|_%max_	fSEsEMG_para%max_ Mean P_di_	fSEsEMG_para%max_ Mean P_oes_	fSEsEMG_para%max_ Mean P_mo_	fSE|sMMG_para_|_%max_ Mean P_di_	fSE|sMMG_para_|_%max_ Mean P_oes_	fSE|sMMG_para_|_%max_ Mean P_mo_
1	0.88	0.90	0.89	0.89	0.89	0.90	0.92
2	0.93	0.94	0.97	0.95	0.89	0.93	0.93
3	0.95	0.87	0.95	0.97	0.82	0.92	0.95
4	0.95	0.92	0.95	0.92	0.94	0.96	0.95
5	0.96	0.96	0.94	0.94	0.96	0.96	0.95
6	0.93	0.88	0.89	0.87	0.92	0.93	0.92
7	0.98	0.98	0.96	0.96	0.97	0.97	0.97
8	0.97	0.98	0.98	0.98	0.96	0.96	0.97
9	0.88	0.91	0.91	0.92	0.92	0.91	0.91
10	0.91	0.82	0.85	0.84	0.91	0.94	0.94
11	0.80	0.76	0.77	0.74	0.96	0.97	0.96
12	0.87	0.91	0.90	0.88	0.91	0.92	0.92
Median (IQR)	0.93 (0.88–0.96)	0.91 (0.88–0.95)	0.92 (0.89–0.96)	0.92 (0.87–0.96)	0.92 (0.90–0.96)	0.94 (0.92–0.96)	0.94 (0.92–0.95)

IQR = interquartile range, P_di_ = transdiaphragmatic pressure, P_oes_ = esophageal pressure, P_mo_ = mouth pressure, fSEsEMG_para__%max_ = fixed sample entropy measurements of surface electromyography of the parasternal intercostal muscles, fSE|sMMG_para_|_%max_ = fixed sample entropy measurements of surface mechanomyography of the parasternal intercostal muscles. All correlations were statistically significant.

**Table 2:** Adjusted R^2^ of the linear and exponential regression models to describe the relationships between measurements of respiratory pressures, fSE|sMMG_para_|_%max_, and fSEsEMG_para%max_.

ID	fSEsEMG_para%max_ fSE|sMMG_para_|_%max_		fSEsEMG_para%max_ Mean P_di_		fSEsEMG_para%max_ Mean P_oes_		fSEsEMG_para%max_ Mean P_mo_		fSE|sMMG_para_|_%max_ Mean P_di_		fSE|sMMG_para_|_%max_ Mean P_oes_		fSE|sMMG_para_|_%max_ Mean P_mo_	
	Lin.	Exp.	Lin.	Exp.	Lin.	Exp.	Lin.	Exp.	Lin.	Exp.	Lin.	Exp.	Lin.	Exp.
1	0.74	0.69	0.81	0.78	0.77	0.72	0.83	0.73	0.80	0.69	0.85	0.75	0.88	0.73
2	0.81	0.80	0.91	0.84	0.96	0.87	0.92	0.82	0.70	0.60	0.76	0.64	0.80	0.65
3	0.86	0.91	0.74	0.81	0.90	0.83	0.94	0.82	0.74	0.76	0.75	0.67	0.79	0.65
4	0.76	0.86	0.91	0.90	0.93	0.89	0.79	0.84	0.81	0.70	0.83	0.67	0.89	0.76
5	0.88	0.91	0.93	0.90	0.91	0.88	0.93	0.86	0.92	0.83	0.90	0.81	0.91	0.79
6	0.91	0.87	0.78	0.74	0.87	0.79	0.75	0.67	0.83	0.78	0.91	0.82	0.83	0.73
7	0.96	0.93	0.95	0.89	0.95	0.88	0.94	0.87	0.95	0.88	0.95	0.87	0.96	0.88
8	0.95	0.86	0.98	0.92	0.98	0.91	0.97	0.90	0.93	0.91	0.94	0.92	0.94	0.92
9	0.84	0.78	0.82	0.71	0.81	0.70	0.86	0.73	0.87	0.79	0.84	0.76	0.89	0.79
10	0.86	0.80	0.74	0.66	0.80	0.74	0.76	0.68	0.84	0.77	0.88	0.85	0.91	0.86
11	0.70	0.74	0.83	0.83	0.73	0.73	0.56	0.57	0.91	0.81	0.95	0.87	0.91	0.87
12	0.73	0.76	0.76	0.81	0.83	0.84	0.82	0.81	0.81	0.71	0.87	0.75	0.88	0.74
Median (IQR)	0.85 (0.75– 0.90)	0.83 (0.77– 0.89)	0.83 (0.77– 0.92)	0.82 (0.76– 0.90)	0.88 (0.80– 0.94)	0.84 (0.74– 0.88)	0.84 (0.77– 0.93)	0.81 (0.71– 0.85)	0.83 (0.80– 0.91)	0.77 (0.71– 0.82)	0.88 (0.84– 0.93)	0.78 (0.71– 0.86)	0.89 (0.85– 0.91)	0.77 (0.73– 0.87)

IQR = interquartile range, Lin. = linear regression model, Exp. = exponential regression model, P_di_ = transdiaphragmatic pressure, P_oes_ = esophageal pressure, P_mo_ = mouth pressure, fSEsEMG_para%max_ = fixed sample entropy measurements of surface electromyography of the parasternal intercostal muscles, fSE|sMMG_para_|_%max_ = fixed sample entropy measurements of surface mechanomyography of the parasternal intercostal muscles.

## Data Availability

The data presented in this study are available from the corresponding author on reasonable request.
